# Removal of Sb(III) by 3D-reduced graphene oxide/sodium alginate double-network composites from an aqueous batch and fixed-bed system

**DOI:** 10.1038/s41598-021-01788-0

**Published:** 2021-11-17

**Authors:** Xiuzhen Yang, Tengzhi Zhou, Renjian Deng, Zhenya Zhu, Atif Saleem, Yuezhou Zhang

**Affiliations:** 1 College of Civil Engineering, Hunan University of Science and Technology, Xiangtan, 411201 China; 2 College of Ecological Health, Hangzhou Vocational and Technical College, Xue Yuan Road. No.68, Hangzhou, 310018 China; 3 Frontiers Science Center for Flexible Electronics (FSCFE), Xi’an Institute of Flexible Electronics (IFE) and Xi’an Institute of Biomedical Materials and Engineering (IBME), Northwestern Polytechnical University, 127 West Youyi Road, Xi’an, 710072 China

**Keywords:** Environmental chemistry, Environmental impact

## Abstract

We created 3D-reduced graphene oxide/sodium alginate double network (GAD) beads to address the problem of local water pollution by antimony. GAD is a novel material with the high specific surface area of graphene and biosecurity of sodium alginate. Due to the introduction of graphene, the thermal stability and specific surface area of GAD are enhanced, as shown from the FTIR, TGA, BET, Raman, and XRD characterizations. The influence of different environmental variables-such as the pH, dosage, temperature, contact time, and sodium chloride concentration on the Sb(III) sorption with GAD-was investigated. The adsorption results fit well with both the pseudo-second order (R^2^ > 0.99) and Freundlich (R^2^ > 0.99) isotherm models. The temperature rise has a negative influence on the adsorption. The Langmuir adsorption capacity is 7.67 mg/g, which is higher than many adsorbents. The GAD results from the fixed-bed adsorption experiment were a good fit with the Thomas model (R^2^ > 0.99). In addition, GAD appears to be a renewable and ideal adsorbent for the treatment of antimony pollution in aqueous systems.

## Introduction

Antimony is an important natural resource, and it is widely used in many industrial products, such as fire retardants^1^, semiconductors^2^, and pigments^3^. China, with its largest reserves, represents almost 80% production of the antimony used globally. However, the environmental problems related with antimony extraction and processing industries become increasingly serious and an alarming concern^4^. In particular, water pollution, where antimony has a high mobility^5^, poses an important risk to human health^6,7^, and the pollutant can be magnified along the food chain^8,9^. Antimony has a different degree of toxicity depending on its oxidation state. Trivalent antimony is more toxic than pentavalent antimony, and different compounds containing antimony also have varying toxicities^10^. High doses of antimony and its derivatives affect the activity of enzymes, causing hypoxia in cells and death^11^.

Adsorption is an effective and feasible method for treating the metal pollution of water^12^. Many materials can be used as adsorbents to treat wastewater, such as zeolites, clay minerals, polymers, and even industrial wastes^13–15^. Graphene and its modified derivatives are potentially ideal adsorbents because of their high specific surface area and excellent mechanical strength^16,17^. Graphene is a honeycomb material that is closely packed in a single layer of carbon atoms, and it generally occurs in a 2D structure^18^. However, the bonding characteristics of graphene (with π–π double bonds and van der Waals force) lead to stacked layers that limit its adsorption performance and applications^19^. Three-dimensional (3D) graphene is a new type of modified graphene that can be assembled by graphene layering and preparation methods, including direct and solution-based synthesis^20^. It not only improves the sorptive ability of metal ions, but also overcomes the problem of graphene layer stacking. There are a number of early reports of metal polluted water being successfully treated by 3D graphene^21,22^. Wu et al. prepared a 3D sulfonated reduced graphene oxide; its adsorption capacity reached 234.8 mg/g for cadmium ions in aqueous solution, which is significantly higher than that of activated carbon (139.9 mg/g) and graphene oxide (23.5 mg/g)^22^.

Sodium alginate is a natural polysaccharide and byproduct of iodine and mannitol extracted from kelp or seaweed. Its molecular structure is composed ofβ-D-mannuronic acid and α-L-guluronic acid^23,24^. Sodium alginate has a strong ability to bond with multi-valence metal ions in aqueous solutions, and it forms a stable hydrogel by cross-linking^25,26^. Its ability to bind to alkali metal ions follows the sequence Mg << Ca < Sr < Ba^27,28^. In addition to applications in food and medicine, sodium alginate is also commonly used as a part of the composite materials that treat water pollution due to its environmental friendliness and cost^29,30^.

The double-network (DN) structure is a hydrogel network consisting of two independent network structures, and it was originally proposed by Gong^31^. One is a poly-electrolyte network structure with high-density cross-linking, and the second is a low-crosslinking or non-cross-linked neutral network^32^. The DN structure gel has a high mechanical strength that is related to an interpenetrating network structure generated by mechanical disruption (e.g., stretching), which causes the first layer of the high cross-linked network to become brittle due to swelling. This swelling causes the initial break at the yield point of the material, and the large number of fractures in the first layer of the network cause the necking phenomenon^31^. The second layer of the network maintains the integrity of the gel by bridging the fractured first layer of the network. Due to the large number of fractures in the first layer of the network, the energy acting on the gel is dissipated, so the mechanical strength of the gel increases through two mutually independent network structures^33,34^. Recently, Yuan et al. enhanced the mechanical strength of hydrogel beads from 0.29 MPa (single-network) to 2.14 MPa (double-network) by forming the double-network structure; the adsorption capacities for Cu^2+^ and Cr_2_O_7_^2-^ were 169.5 mg/g and 72.46 mg/g, respectively^35^. This result indicates that double-network structure materials are feasible adsorbents for dealing with water pollution caused by metals. However, well defined 3D reduced graphene based double network hydrogel with sodium alginate have seldom been reported. Moreover, the adsorption performance of GAD under different application scenario such as fixed bed column hardly been reported before.

Other research focused on the potential of combining high specific surface area graphene-derived materials^36–38^ with low toxicity sodium alginate^39,40^, which were combined by the sol–gel method and the double-network hydrogel beads. The 3D graphene structure was prepared by reduction reactions and graphene self-assembly in a hydrothermal environment^41,42^. The synthesis method reduces the issue of the graphene layer stacking and increases the performance in the solid–liquid separation with excellent adsorption for wastewater treatment. We specifically focused on testing its application to critical water pollution problems in the environment, such as antimony-related industrial and mining enterprises in central south China (reference indicating case study and location).

## Materials and methods

### Preparation of 3D-reduced graphene oxide/sodium alginate double-network hydrogel beads

Graphene oxide was provided by Najing Jicang Nano Technology Co., Ltd. Since the trivalent form of antimony is 10 times more toxic than pentavalent antimony in aqueous systems^43^, this was the key target ion. The antimony test solution was prepared by dissolving potassium antimony tartrate semihydrate (KSbOC_4_H_4_O_6_·1/2H_2_O, AR, Xilong Scientific Co., Ltd., China) in deionized water (the oxidation of trivalent antimony is considered unlikely under experimental conditions^44^). The pH of the solution was adjusted using hydrochloric acid and sodium hydroxide in all experiments. The sodium alginate was chemically pure and purchased from Sinopharm Chemical Reagent Co., Ltd., China. Anhydrous calcium chloride (CaCl_2_) was analytically pure and obtained from the Tianjin Fengchuan Chemical Reagent Technologies Co., Ltd., China. The ascorbic acid was analytically pure and provided by Xilong Scientific Co., Ltd., China. Analytically pure absolute ethyl alcohol was purchased from Tianjing Fuyu Co., Ltd., China.

Sodium alginate single-network hydrogel beads (SAS) were prepared. Sodium alginate (2 g) was added into water (100 mL) with magnetic stirring for 1 h. Then, the sodium alginate solution was dripped into a 5 wt% solution of CaCl_2_ under neutral conditions. The calcium ions and sodium alginate formed hydrogel beads, and the surface moisture was dried with absolute ethyl alcohol.

Graphene oxide (200 mg) was dispersed into deionized water (100 ml) in an ultrasonic bath. Sodium alginate (2 g) was added into the solution of graphene oxide and mixed with magnetic stirring for 1 h. Then, the graphene oxide and sodium alginate mixture was dripped into a 5 wt% solution of CaCl_2_ under neutral conditions. The calcium ions and sodium alginate formed hydrogel beads by cross-linking. The GO roughly accounted for 10 wt% of the prepared beads. The hydrogel beads were then immersed into an ascorbic acid solution in a thermostat water bath at 90 ℃ for 8 h. The 3D graphene structure was formed through hydrothermal reactions due to the graphene self-assembly effect forming the double network structure. Lastly, the 3D-reduced graphene oxide double-network hydrogel bead (GAD) was prepared after the surface moisture was removed.

### Characterization methods

The specific surface area, total pore volume, and pore size distribution were determined by nitrogen adsorption/desorption at 77.4 K using the ASAP 2020 (Micromeritics, USA), and all the beads were degassed at 299 K before the measurements. The thermo gravimetric analysis (TGA) was carried out under a nitrogen atmosphere on a Netzsch STA449F3 (Germany) in the temperature range from room temperature to 600 ℃ with a heating rate of 10 ℃/min. Scanning electron microscope (SEM) images were captured by a JSM-6390LV (Japan). Fourier-transform infrared spectroscopy (FT-IR) spectra were recorded on a Perkin Elmer Spectrum 100 spectrometer (USA). Raman spectroscopy spectra were recorded on a Thermo Fisher Scientific DXR (USA). X-Ray Diffraction was tested by D8 Advance Bruker (Germany) in the scope of 5°–90°. The atomic absorption spectra were acquired by an atomic absorption spectrophotometer (AA-7003, East and West Analytical Instruments, Inc., China) to analyze Sb(III). All the samples were filtered through a 0.45 μm filter membrane. The adsorption study was performed in a water bath constant temperature vibrator (THZ-82, Jintan Ronghua Instrument Manufacture Co., Ltd., Jiangsu, China), and the antimony-containing solution was transferred by the BT600-2 J (Baoding Longer pump Co., Ltd., Baoding, China) in the fixed-bed adsorption experiment. Suitable replicates and analytical blanks were measured to ensure analytical reliability.

### Adsorption experiment

#### Batch experiment

The GAD hydrogel beads were used in subsequent adsorption experiments. The effect of the adsorption of different experimental parameters, such as the initial pH, dosage of the adsorbent, contact time, temperature, and the initial concentration of Sb(III) were investigated. The required number of beads was added into a 100-ml polyethylene bottle containing 50 ml of Sb(III) solution. The pH was adjusted using 0.1 M HCl and NaOH solution, and the reaction was allowed to proceed in an incubator shaker at a fixed temperature (298 K) and rotation speed (150 rpm). The residual Sb(III) in solution was monitored by an atomic absorption spectrophotometer after an appropriate amount of contact time.

The adsorption kinetics experiment was carried out at pH 6. A portion of the beads (specify the mass added) was added into the solution with Sb(III) and left to react in the incubator shaker. The adsorption capacity was determined from the residual Sb(III) concentration in solution and monitored at time points of 15, 30, 60, 120, 180, 240, 300, 360, 480, 600, and 720 min. We mimicked the waste-water release characteristics from mining activities by carrying out the adsorption isotherm experiment with a dose of 400 mg beads (dry weight) and the initial concentration of the Sb(III) solution in the range of 10–220 mg/L.

The dose–response experiment was conducted at pH 6. A 50 mL portion of the Sb(III) solution with the initial concentration of 10 mg/L was added into six 100-ml polyethylene bottles. The adsorption reaction was carried out in the dose range of 50–600 mg (dry weight) in the incubator shaker (298 K) for 240 min in duplicate.

To investigate the adsorption kinetics of the hydrogel, 200 ml of the aqueous Sb(III) solution with initial concentrations of 10 mg/L, 20 mg/L, and 50 mg/L was prepared ,and the pH was adjusted to 6. A 1600 mg portion of adsorbent (dry weight) was added into these solutions. The reaction was carried out at 298 K and agitated at 150 rpm; the residual concentrations of Sb(III) were monitored over time.

Reusability represents an important factor for treatment systems and the performance of adsorbents, and we investigated the adsorption–desorption reliability of GAD. Reuse of the GAD hydrogel was evaluated using 0.1 mol/L HCl as the regeneration agent. The initial concentration of Sb(III) was 10 mg/L and 400 mg of GAD (dry weight). After adsorption at pH 6, the Sb(III)-loaded GAD was recovered, treated with CaCl_2_ for 240 min, washed with deionized water, and used in further adsorption–desorption experiments. This process was repeated for five cycles.

#### Column adsorption

Although batch adsorption studies provided useful information on the application of adsorption for the removal of specific waste constituents, column studies can provide basic data on the systematic process, which is essential for the scale-up for engineering applications in wastewater treatment.

The fixed-bed column experiment was carried out in a polymethyl methacrylate column with an inner diameter of 2 cm and a length of 50 cm. Appropriate quantities of the adsorbent and an inert sand support were then added into the column and saturated using water. The Sb(III) solution was injected into the column in an upward flow direction using a peristaltic pump. The migration velocity was adjusted, and the bed depth was confirmed. The samples were taken at 5, 15, 30, and 60 min and subsequently every 60 min. The concentration of Sb(III) in the solution was then measured by the AAS to determine the breakthrough curve of Sb(III) under different conditions. All experiments were carried out in triplicate.

## Results and discussion

### The characteristics of GAD

**Figure 1:**
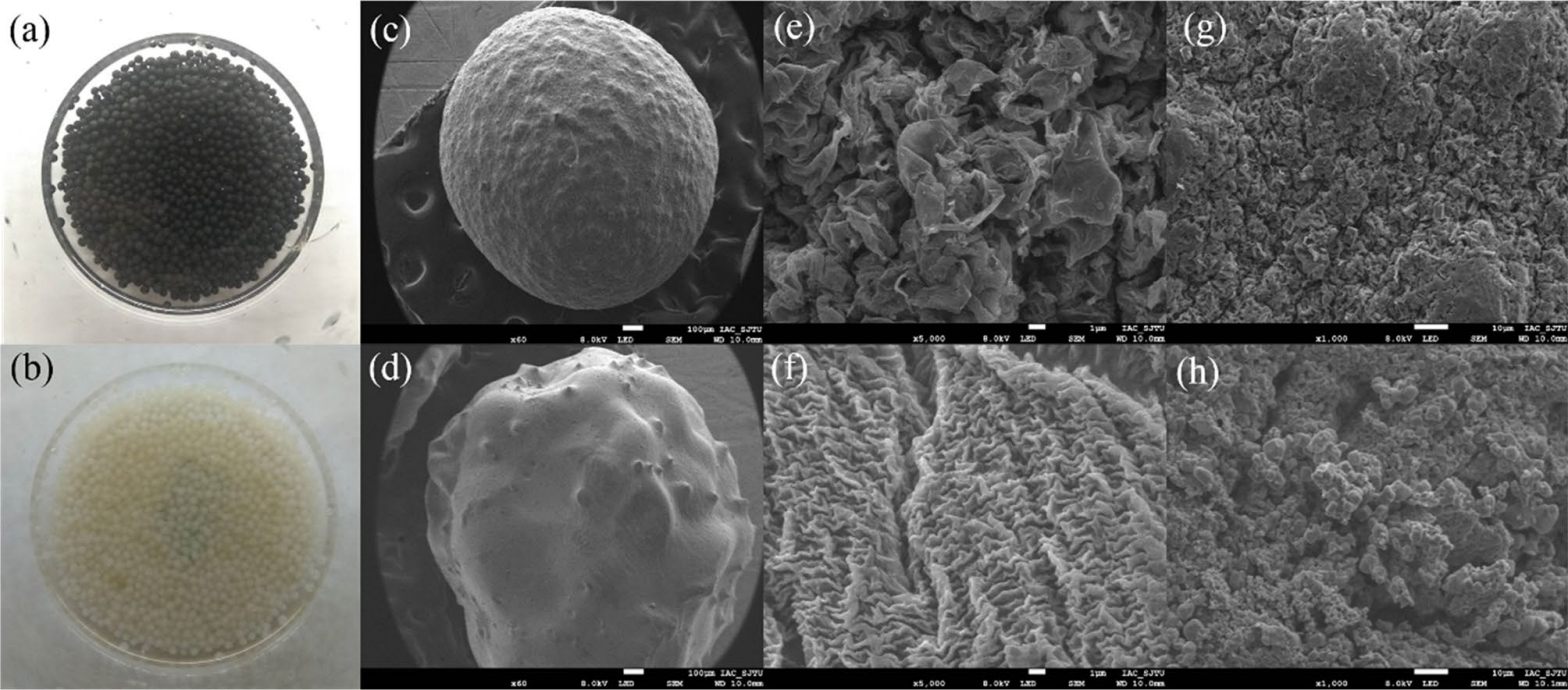
Photographs of (**a**) GAD and (**b**) SAS, (**c**), (**e**), and (**g**) are SEM images of GAD, (**d**) and (**f**) are SEM images of SAS; and (**h**) SEM images of GAD after adsorption.

The SEM images are shown in Fig. 1; GAD is the black hydrogel bead (Fig. 1a) and SAS is the yellow-white hydrogel bead (Fig. 1b). The graphene layers are clearly seen due to the presence of the graphene oxide (Fig. 1e), which indicates that the GAD hydrogel beads have been successfully prepared. Graphene layers are inclined to curl, which is caused by the van der Waals force and π–π stacking between the graphene layers^45,46^, therefore, the GAD beads exhibited undulating features. In contrast, there are no obvious graphene layers and a relatively smooth surface in the SAS after freeze-drying. This result does not support the stability of the SAS bead structure in the aqueous solution, making it easy for the beads to swell. Compared to the single-network structure of the SAS, the 3D structure formed through graphene self-assembly under hydrothermal conditions presents a distorted and interwoven structure of graphene layers and the hydrogel^35,47^. This structure is useful because it improves the mechanical strength of GAD and supports a high porosity, which helps to increase the adsorption performance. After adsorption (Fig. 1h), the pore structure of the GAD is reduced, an amorphous phase is attached to the surface, probably from the Sb(III) solution. We speculate that after the adsorption reaction, the adsorbed Sb(III) is oxidized due to the drying treatment, adhering to the surface of GAD in the form of an amorphous solid.

**Figure 2:**
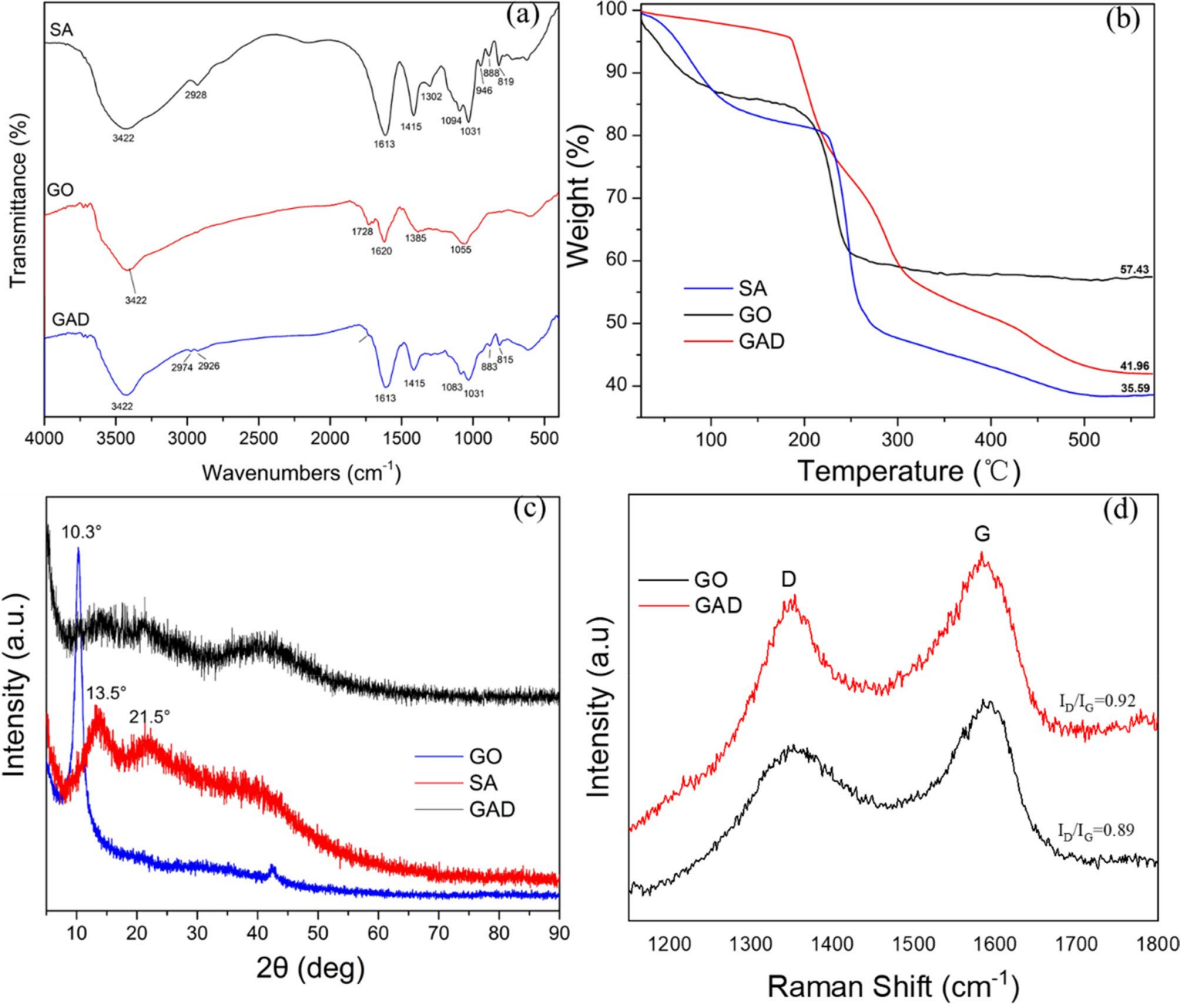
(**a**) FTIR spectra of SA, GO and GAD, (**b**) TGA of SA, GO, and GAD, (**c**) XRD of SA, GO, and GAD, (**d**) Raman spectra of GAD and GO.

The analysis by FTIR is shown in Fig. 2a, the features at 3422 cm^−1^, 2928 cm^−1^, 1613 cm^−1^, 1728 cm^−1^, and 1055 cm^−1^ are characteristic peaks caused by –OH, –CH_2_–, C = C, C = O, and C–O–C stretching vibrations, respectively. The peaks at 1094 cm^−1^ and 1031 cm^−1^ are likely due to the C–O–C stretching vibration. The characteristic peak of 1385 cm^−1^ is led by the methyl bending vibration. Generally, organic compounds are more sensitive in the mid-infrared region (4000–600 cm^−1^) and no characteristic peaks appear or disappear after preparation (with some peak shifts) because of the sodium alginate cross-linked calcium ions^48^. In the spectrum of GAD, two characteristic peaks at 2974 cm^−1^ and 1726 cm^−1^ are affected when GO is introduced (compared to the similar SAS spectrum), with a blue shift at 1085 cm^−1^, a red shift at 2931 cm^−1^, 1617 cm^−1^, 1430 cm^−1^, 1085 cm^−1^, 892 cm^−1^, and 822 cm^−1^ to different degrees. Beyond that, the preparation of GAD was considered to have been effective. After adsorption, five characteristic peaks at 3422 cm^−1^, 1613 cm^−1^, 2974 cm^−1^, 1031 cm^−1^, and 883 cm^−1^ showed a blue shift, with two characteristic peaks at 2926 cm^−1^ and 1415 cm^−1^ showing a red shift; one characteristic peak at 1083 cm^-1^ disappeared. Therefore, the adsorption process was dominated by a physical process.

The TGA analysis is shown in Fig. 2b, and the decrease in the gross weight was due to the moisture evaporation in Stage 1 (room temperature–200 ℃)^49^. In the second stage, the total weight loss at 200 to 300 °C was due to the high temperature cracking of the oxygen functional groups^48^. The graphene oxide material has a good thermal conductivity, and thus the thermal conductivity of GAD correspondingly increased. In comparison, the initial temperatures of the second stage for sodium alginate and GO are 230 °C and 215 °C, respectively. The second stage of GO occurs at a lower temperature than for sodium alginate due to the higher thermal conductivity of GO. Therefore, the thermal conductivity of GAD also increased. The weight loss in the third stage, from 300 to 500 °C, was mainly due to the decomposition of the carboxyl groups and the release of CO_2_^50^. Over this period, the weight of GO did not decrease significantly, in contrast to the significant decrease in the weight for SAS and GAD. In the final stage, the mass percentages of GO, GAD, and SAS were 57.43%, 41.96%, and 35.59%, respectively. The thermal stability of the composite material was therefore enhanced.

The characterization of GO, sodium alginate, and GAD by XRD is presented in Fig. 2c. For GO, a sharp diffraction pattern shows characteristic diffraction peaks at 2θ = 10.3°^51^. In the case of SA, a less ordered structure can be seen, but relatively strong characteristic peaks are observed at 2θ = 13.5° and 2θ = 21.5°^35^. For GAD, 2, characteristic peaks at 13.5° and 21.5° still exist, but the typical characteristic peak of GO disappears at 10.3°. According to previous research^52^, this phenomenon occurs due to the 3D self-assembly reaction of GO under the influence of high temperatures and the hydrothermal reduction reaction, as well as the ordered stacking of graphene layers.

The Raman spectrum provides additional information on the behavior of the carbon structure of the material during the preparation process. As shown in Fig. 2d, the characteristic peaks for GAD and GO are at 1351 cm^−1^ and 1593 cm^−1^, respectively^53^. The peak D is predominantly due to the vibration of sp^3^ carbon atoms caused by the defects or disorders in the carbon structure. The peak G is mainly due to the in-plane stretching vibration of carbon atoms from the sp^2^ hybridization^54^. In the GAD spectrum, the peak G shifted from 1593 to 1583 cm^−1^, and the intensity ratio of I_D_/I_G_ changed slightly and increased from 0.89 to 0.92, which indicates an decreased degree of graphitization in the materials^53^ due to the synthesis process that introduced structural defects and increased the disorder of the materials^55^.

**Figure 3:**
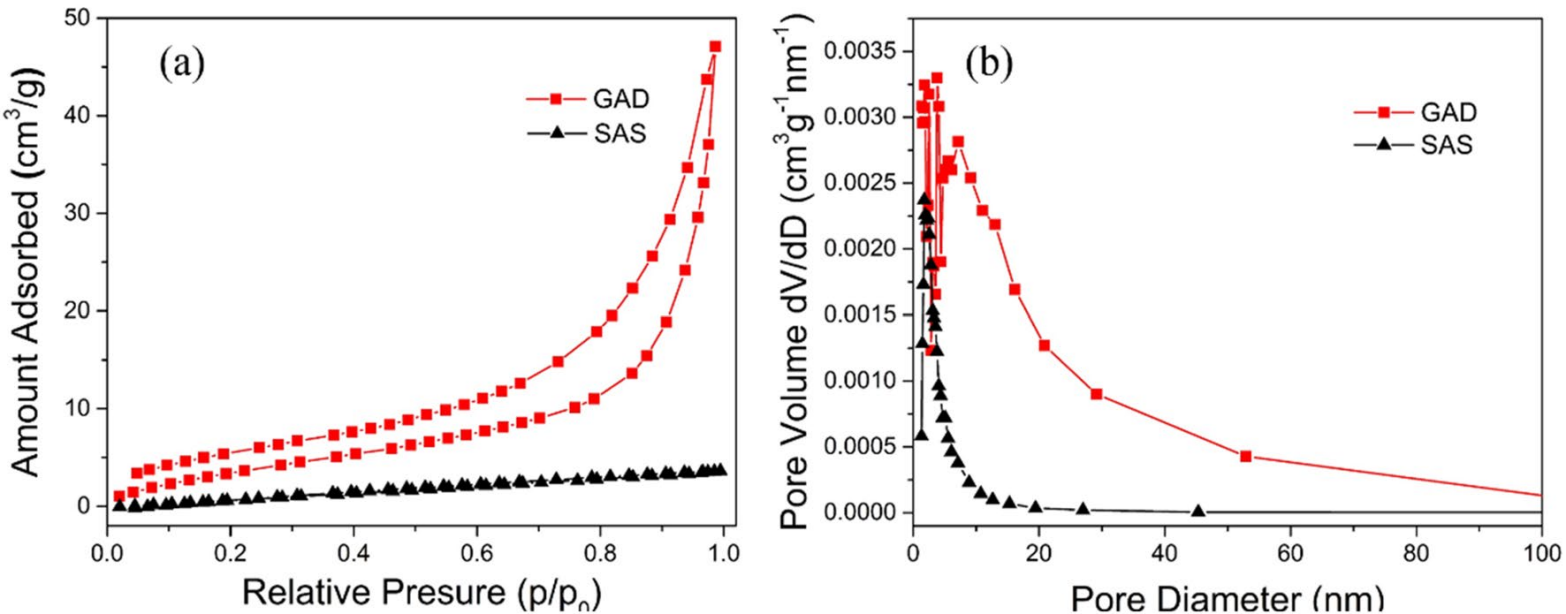
(**a**) N_2_ adsorption and desorption isotherm and (**b**) pore size distributions of GAD and SAS.

Generally, as the specific surface area increases for solid materials, the adsorption performance becomes stronger^49^. According to the results of the specific surface area and pore size analysis (Fig. 3, Table 1), the specific surface areas of GAD and SAS are 29.8 m^2^/g and 10.1 m^2^/g, respectively. The specific surface area of the GAD increased from 10.1 to 29.8 m^2^/g by the introduction of GO, and for GAD, it is nearly three times higher than for SAS. The total pore volume is 0.076 cm^3^/g and 0.005 cm^3^/g, which increases the capacity for pollutant uptake. Due to the formation of the double network and the introduction of GO, the distribution of the pore diameter increases for the mesopores’ size range from 5 to 50 nm, and the average pore diameter increases from 1.3 to 1.8 nm.

**Table 1:** The physical properties of GAD and SAS.

Characteristics	GAD	SAS
Specific surface area (m^2^/g)	29.8	10.1
Average pore diameter (nm)	1.8	1.3
Total pore volume (cm^3^/g)	0.076	0.005

### Adsorption experiment

The removal efficiency (%) for Sb(III) was calculated using Formula (1), and the adsorption capacity was calculated using Formula (2) in the adsorption experiment.

1 $$ \% removal = \frac{{C_{0} - C_{e} }}{{C_{0} }} \times 100 $$

2 $$ {\text{q}}_{{\text{e}}} = \frac{{(C_{0} - C_{{\text{e}}} ) \cdot V}}{{\text{m}}} $$

where C_0_(mg/L) is the initial concentration of Sb(III) in solution, C_e_(mg/L) is the Sb(III concentration at equilibrium, V(L) is the volume of the solution, and m(g) is the weight of the adsorbent.

**Figure 4:**
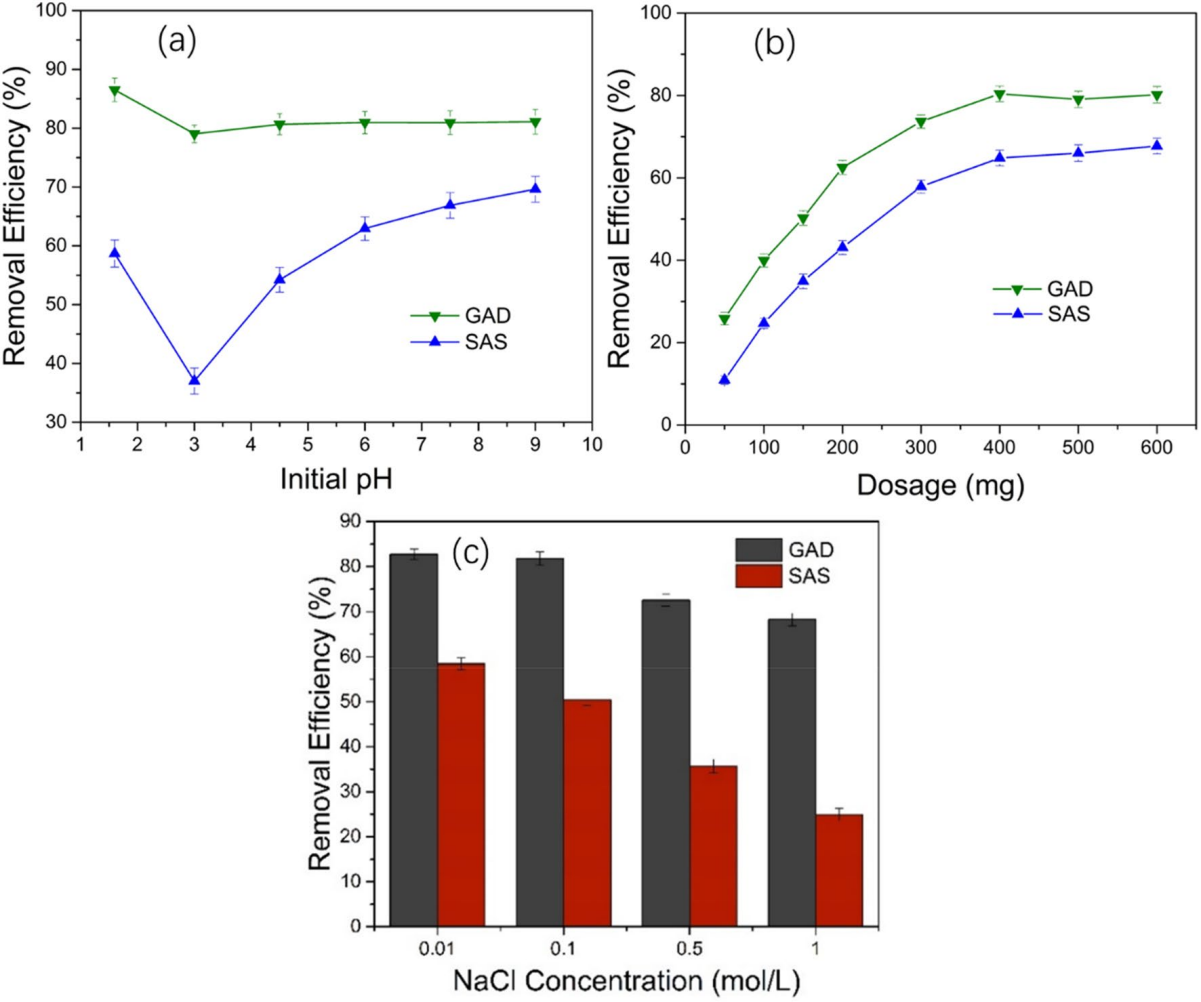
Effects of (**a**) the initial pH on the adsorption efficiency of Sb(III) (dosage = 400 mg/50 mL dry weight; Sb(III) concentration = 10 mg/L; temperature = 298 k; rotation speed = 150 rad/min; contact time = 240 min), (**b**) the GAD dose on adsorption efficiency of Sb(III) (pH = 6; Sb(III) concentration = 10 mg/L; temperature = 298 k; rotation speed = 150 rad/min; contact time = 240 min), (**c**) the NaCl concentration on adsorption efficiency of Sb(III) (pH = 6; dosage = 400 mg/50 mL dry weight; Sb(III) concentration = 10 mg/L; temperature = 298 k; rotation speed = 150 rad/min; contact time = 240 min).

As shown in Fig. 4a, the removal efficiency of GAD for Sb(III) reached 86% in solution at pH 1.6, but for SAS, it was only 58% under the same experiment conditions. The increase in the pH results in the removal efficiency of the 2 adsorbents of 79% and 37% when the pH is 3, and the removal efficiency of GAD is double that of SAS. The efficiency of GAD gradually stabilizes above pH 3, with the adsorption efficiency at 81% at pH 6. The adsorption performance of SAS changes significantly with the pH, rising rapidly from a minimum at pH 3 to about 69% at pH 9. In the solution environment of low pH, antimony is predominantly found as SbO^+^ and HSbO_2_^56^. Both GO and SA are protonated under acidic conditions, creating a positive charge on the surface through the formation of –COOH_2_^+^ and –OH_2_^+^^57^, which enhances the adsorption of neutral antimony ions such as HSbO_2_. The dissolution of nonstructural calcium ions in the adsorbents results in the increase in the adsorption sites. Thus, the adsorbents have a better removal efficiency at a low pH. The positive charge of the adsorbents decreases when the pH increases from 1.6 to 3, and the electrostatic adsorption force weakens gradually; the solubility of the nonstructural calcium ions decreases, and the adsorption site decreases. Therefore, there is a decrease in the adsorption performance. When the pH rises above 3, antimony exists mainly in HSbO_2_, the negative charge of the adsorbents was gradually enhanced, and the electrostatic adsorption favors Sb(III).

We investigated the effect of different doses of adsorbent (50–600 mg/50 mL dry weight) on the removal efficiency of Sb(III) in solution (Fig. 4b). The removal efficiency for both GAD and SAS was significantly enhanced with the increasing dose. The removal efficiency of GAD is 26% at a dose of 50 mg and increases to a maximum at the 400 mg dose (80%), reaching the adsorption equilibrium. The removal efficiency is significantly higher than SAS. The relationship between the adsorption sites and removal efficiency has a positive correlation^58^. Two reasons may account for this phenomenon. First, the adsorbent dose increase results in the adsorption site increase. Second, the opportunity for the interaction of antimony ions with adsorbents also increases^59^. There is no significant change in the removal efficiency in the doses above 400 mg, and the adsorption performance of GAD is still significantly better than that for SAS.

Sodium chloride is a common inorganic salt in natural water. The experiment to investigate the effect of different NaCl concentrations on the adsorption performance was carried out across solution concentrations of NaCl of 0.1 to 1 mol/L. As shown in Fig. 4c, as the NaCl concentration increases, the adsorption of Sb(III) decreases due to the competition between the sodium and calcium ions. The removal efficiency of SAS-adsorbed antimony ions is 25% at 1 mol/L NaCl. However, when the NaCl concentration is 0.01–0.1 mol/L, the GAD removal efficiency is still about 80%, which slightly decreases when the NaCl concentration increases. The removal efficiency is 68% at the NaCl concentration of 1 mol/L. Above that, the adsorption stability and adsorption performance of the adsorbent improved after the GAD was prepared through the introduction of GO.

### Adsorption kinetics

The study of the kinetic characteristics in the use of GAD hydrogel beads to remove Sb(III) in aqueous solution is beneficial to the practical application of the adsorbents in wastewater treatment systems and for understanding the adsorption mechanism^60^. The fit of experimental data to the models of the pseudo-first-order, pseudo-second-order, and intraparticle diffusion was assessed. This evaluation was based on the following formulae^61^:

3 $$ \ln (q_{e} - q_{t} ) = \ln q_{e} - k_{1} t $$

4 $$ \frac{t}{{q_{t} }} = \frac{1}{{k_{2} q_{e}^{2} }} + \frac{1}{{q_{e} }}t $$

5 $$ q_{t} = k_{i} t^{1/2} + a $$

where q_e_ (mg/g) is the adsorbent Sb(III) quantity at equilibrium, q_t_ (mg/g) is the adsorbent quantity at time t, and k_1_ (min^−1^) and k_2_ (g mg^−1^ min^−1^) are the first-order and second-order equilibrium rate constants, respectively. k_i_ is the adsorption efficiency constant of the intraparticle diffusion model.

**Figure 5:**
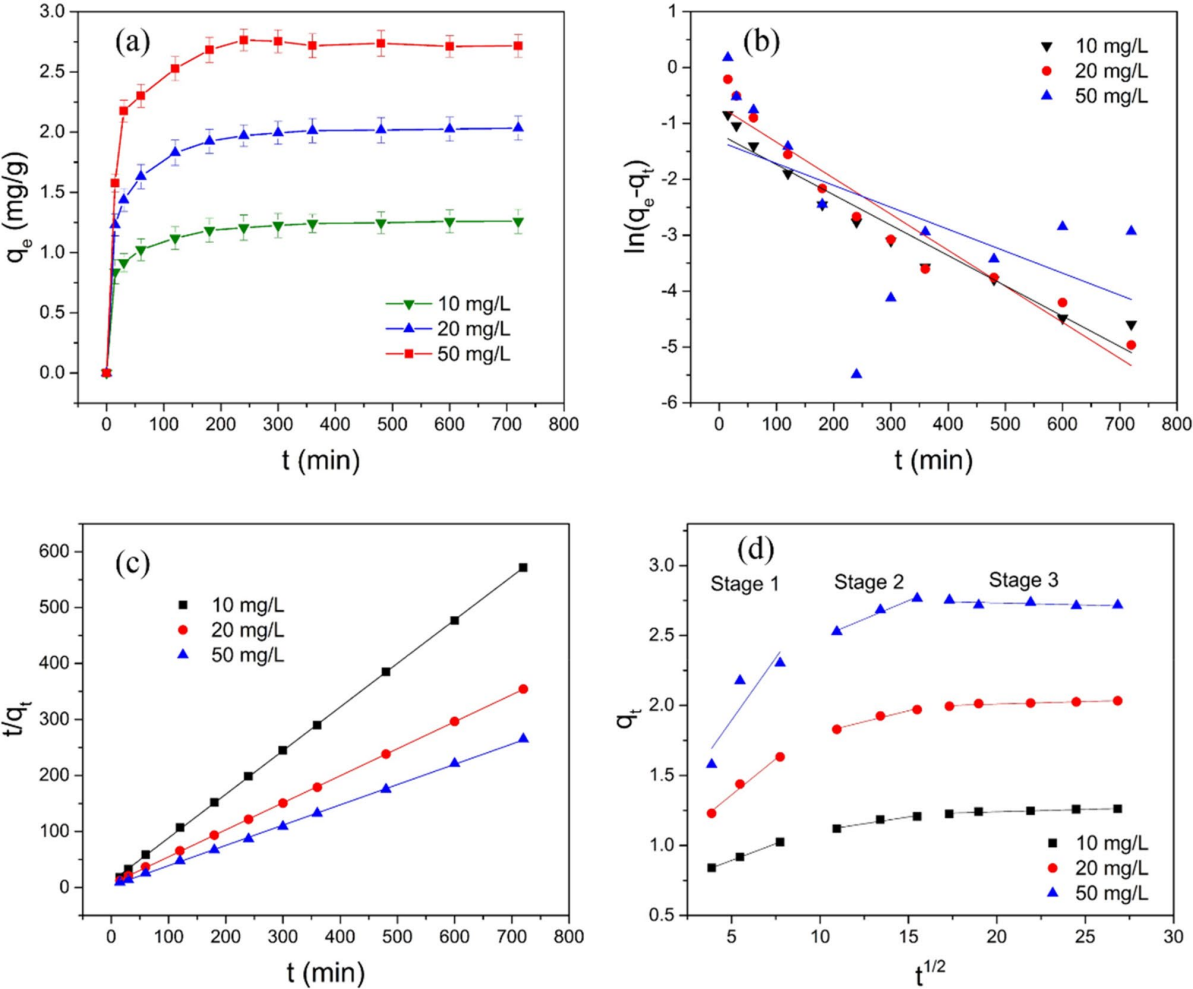
(**a**) Effect of contact time on the removal of Sb(III) by GAD. (**b**) Pseudo-first-order reaction model. (**c**) Pseudo-second-order reaction model. (**d**) Intraparticle diffusion model (pH = 6; dosage = 1600 mg/200 mL dry weight; Sb(III) concentration = 10, 20, 50 mg/L; temperature = 298 k; rotation speed = 150 rad/min; contact time = 720 min).

For the pseudo-first-order, the pseudo-second-order, and the intraparticle diffusion models, ln(q_e_–q_t_) to time(t), t/q_t_ to time, and q_t_ to t_1/2_ were plotted (Fig. 5b–d), and a linear fit was derived.

As shown in Fig. 5a, both the contact time and the initial concentration of the adsorption reaction affect the adsorption performance of the adsorbent. In the initial stage of the reaction, the reaction and adsorption capacity increase rapidly due to the high adsorption sites and high concentration of pollutants^62^. As the contact time increased, the reaction rate slowed and arrived at the adsorption equilibrium at 240 min. The adsorption capacity was also enhanced with the increased concentration of Sb(III)^63^.

The pseudo-first-order model (Fig. 5b) and pseudo-second-order model (Fig. 5c) were applied to the described reaction process of the GAD adsorption of Sb(III) under different conditions. At high concentrations of Sb(III) (50 mg/L), the pseudo-first-order model did not accurately describe the adsorption reaction (R^2^ is 0.3067). However, the pseudo-second-order model fits well with the data (Table 2) on the adsorption at different concentrations (R^2^ above 0.99). According to the pseudo-second-order model fitting results, the theoretic adsorption capacities are 1.28 mg/g, 2.07 mg/g, and 2.76 mg/g, and there is only minor a difference with the actual adsorption capacity (1.27 mg/g, 2.04 mg/g and 2.77 mg/g).

**Table 2:** The parameters of the adsorption kinetic study.

The initial concentration of Sb(III) (mg/L)	10	20	50
q_exp_ (mg/g)	1.27	2.04	2.77
**Pseudo-first order**			
q_cal_ (mg/g)	0.31	0.51	0.27
k_1_	0.0054	0.0064	0.0039
R^2^	0.9441	0.9353	0.3067
**Pseudo-second order**			
q_cal_ (mg/g)	1.28	2.07	2.76
k_2_	0.0581	0.0366	0.0491
R^2^	0.9999	0.9999	0.9996
**Intraparticle diffusion**			
k_i1_	0.0477	0.1028	1.0040
k_i2_	0.0194	0.0314	0.0529
k_i3_	0.0034	0.0036	− 0.0030
R_1_^2^	1.0000	0.9859	0.8027
R_2_^2^	0.9526	0.9762	0.9836
R_3_^2^	0.8938	0.9011	0.4452

Figure 5d shows the intraparticle diffusion model was applied to further investigate the adsorption kinetics of the Sb(III) sorption to GAD, which is divided into 3 stages: an initial steep slope, showing the transport of Sb(III) from the aqueous solution to the adsorbent surface through diffusion (concentration gradient), the diffusion of Sb(III) into the pores of the adsorbent, which is rate-determining and slower, and the equilibrium process, where the Sb(III) ions are adsorbed on sites on the internal surface of the pores and intraparticle diffusion begins to slow down until equilibrium is reached. The intraparticle diffusion model does not pass through the origin and the intercept of the equation increases with the increase in the initial concentration, which indicates the intraparticle diffusion is not the only controlling factor.

### Adsorption isotherm

**Figure 6:**
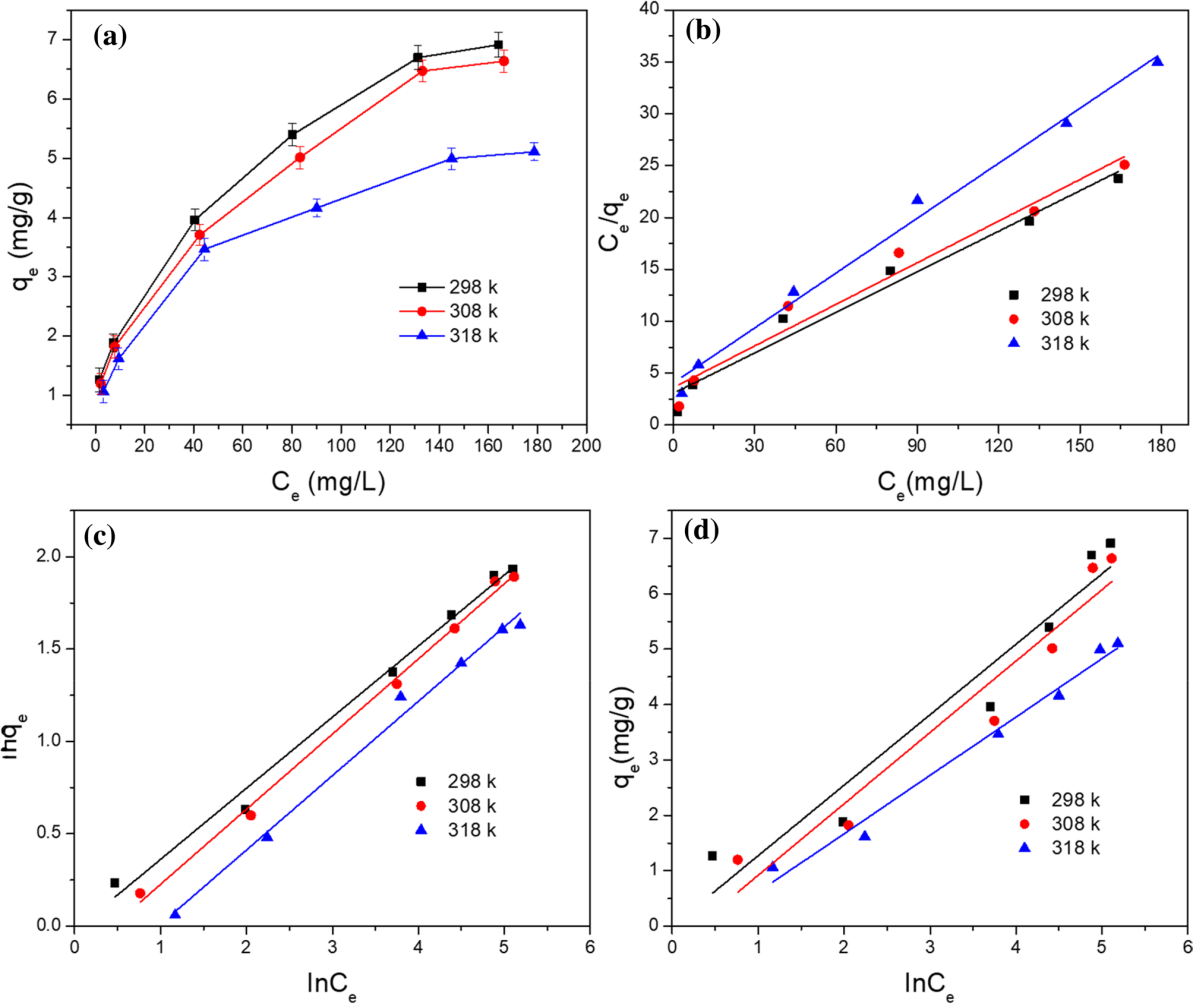
(**a**) Effect of temperature on the removal of Sb(III) by GAD. (**b**) Langmuir isotherm fitting. (**c**) Freundlich isotherm fitting. (**d**) Temkin isotherm fitting (pH = 6; dosage = 400 mg/50 mL dry weight; Sb(III) concentration = 10–220 mg/L; temperature = 298, 308, 318 k; rotation speed = 150 rad/min; contact time = 240 min).

The initial Sb(III) concentrations (10–220 mg/L) were prepared and reacted with the adsorbents in an incubator shaker (150 rpm) for 240 min at pH 6 at the temperatures of 298 K, 308 K, and 318 K. The dose of GAD was 400 mg/50 ml. The Langmuir, Freundlich, and Temkin isotherm models were applied to fit the experiment data collected. These are described below.

6 $$ \frac{{C_{e} }}{{q_{e} }} = \frac{{C_{e} }}{{q_{\max } }} + \frac{1}{{q_{\max } K_{L} }} $$

7 $$ \ln q_{e} = \ln K_{F} + \frac{1}{n}\ln C_{e} $$

8 $$ q_{e} = B\ln A + B\ln C_{e} $$

where C_e_ (mg/L) is the mass concentration of Sb(III) when the system is at equilibrium. K_L_ (L/mg) is the Langmuir adsorption constant. K_F_ (L/mg) is a Freundlich constant related to the adsorption capacity and 1/n is an empirical parameter indicating the favorability of the adsorption^64^. It is assumed that when 1/n is larger than 2.0, the material is difficult to adsorb. A is the Temkin adsorption isotherm binding constant, and B is the constant related to the heat of adsorption. It assumes that linearly decrease of adsorption heat as the Sb(III) covers the surface of GAD, and binding energies of Sb(III) will be characterized up to maximum. As shown in Fig. 6a, the adsorption capacity of GAD beads for Sb(III) is enhanced and the adsorption reaction rate is reduced with an increase in the initial Sb(III) concentration. The adsorption capacity of GAD is 1.06 mg/g for an initial concentration of 10 mg/L at 298 k, rising to 6.91 mg/g for an initial concentration of 220 mg/L. In addition to the initial sorbate, the temperature also affects the adsorption reaction. Since the adsorption decrease with increase in temperature and desorption of molecule occurs from GAD surface at higher temperature. This could be due to week interaction between adsorbent surface and adsorbate ions, which supports the physical adsorption. In this reaction, increasing the temperature reduces the adsorption capacity, therefore, this adsorption process is probably an exothermic reaction^15,65,66^.

The Langmuir (Fig. 6b), Freundlich (Fig. 6c), and Temkin (Fig. 6d) adsorption isotherm models were applied to investigate the thermodynamic characteristics of the adsorption reaction for GAD. The Langmuir (R^2^ > 0.96) and Freundlich (R^2^ > 0.99) adsorption isotherms had a good fit (Table 3) to the experimental data collected at different reaction temperatures (298 K, 308 K, and 318 K). The maximum adsorption capacity was 7.67 mg/g at 298 k. The Freundlich constant 1/n is 0.3851, 0.4096, and 0.4026 for the different temperatures, but still between 0.1–0.5 and higher correlation coefficient as compared to the Langmuir isotherm, which showed the favorable multilayer, heterogonous adsorption on the adsorbent. This parameter has a strong relationship with the adsorption reaction, with the variation in the value reflecting the homogeneity of the adsorbent surface^13^. The Temkin adsorption isotherm also produced a good fit with the reaction process (R^2^ > 0.93), which indicates the interaction between Sb(III) and GAD is favored because of availability of a large number of active sites. However, the physical interaction of adsorbent and adsorbate weekend at elevated temperature because of van der waal and hydrogen bond weakening.

**Table 3:** Parameters of the isotherm models.

T(K)	Langmuir			Freundlich			Temkin			
	q_max_ (mg/g)	K_L_	R^2^	K_F_	1/n	R^2^	A	B		R^2^
298	7.67	0.0431	0.9732	0.9768	0.3851	0.9915	0.9987	1.2728		0.9351
308	7.47	0.0372	0.9665	0.8348	0.4096	0.9960	0.7503	1.2890		0.9372
318	5.66	0.0437	0.9914	0.6751	0.4026	0.9921	0.6595	1.0533		0.9839

The maximum adsorption capacity of the GAD beads is compared to that of natural materials, artificial adsorbents, bio-based adsorbents, and graphene-based adsorbents for Sb(III) in Table 4. Even considering methodological variation, the GAD has a better adsorption performance compared to other non-graphene-based adsorbents with only a minor proportion of graphene (about 10 wt% dry weight).

**Table 4:** Comparison of the adsorption capacity of Sb(III) onto various adsorbents.

Adsorbent	Initial concentration (mg/L)	Adsorption capacity (mg/g)	pH	References
Multi-walled carbon nanotube	4	0.33	7	^15^
Bentonite	4	0.56	6	^14^
Mycrositis	500	4.88	4	^67^
Fe_2_O_3_-modified nanotube	3.2	6.23	7	^68^
PVA-Fe^0^ granules	20	6.99	7	^69^
Cyanobacteria Synechocystis sp.	100	4.68	7	^70^
**GAD**	220	7.67	6	This study
Graphene	10	8.05	11	^57^

The thermodynamic parameters were calculated using the equations shown below:

9 $$ \Delta G = - RT\ln K_{D} $$

10 $$ \Delta G = \Delta H - T\Delta S $$

11 $$ \ln K_{D} = \frac{\Delta S}{R} - \frac{\Delta H}{{RT}} $$

12 $$ K_{D} = \frac{{C_{0}  - C_{t} }}{{C_{t} }} \times \frac{V}{m} $$

where K_D_ is the thermodynamic equilibrium constant; ΔG is the free energy of adsorption, kJ/mol; ΔH is the enthalpy of adsorption, kJ/mol; ΔS is the entropy of adsorption, J/(mol·K); R is the ideal gas constant, 8.314 J/(mol·K); T is the temperature in the adsorption. C_0_ (mg/L) is the initial concentration of Sb(III). Ce (mg/L) is the concentration of Sb(III) at equilibrium.

### Thermodynamic parameters

**Figure 7:**
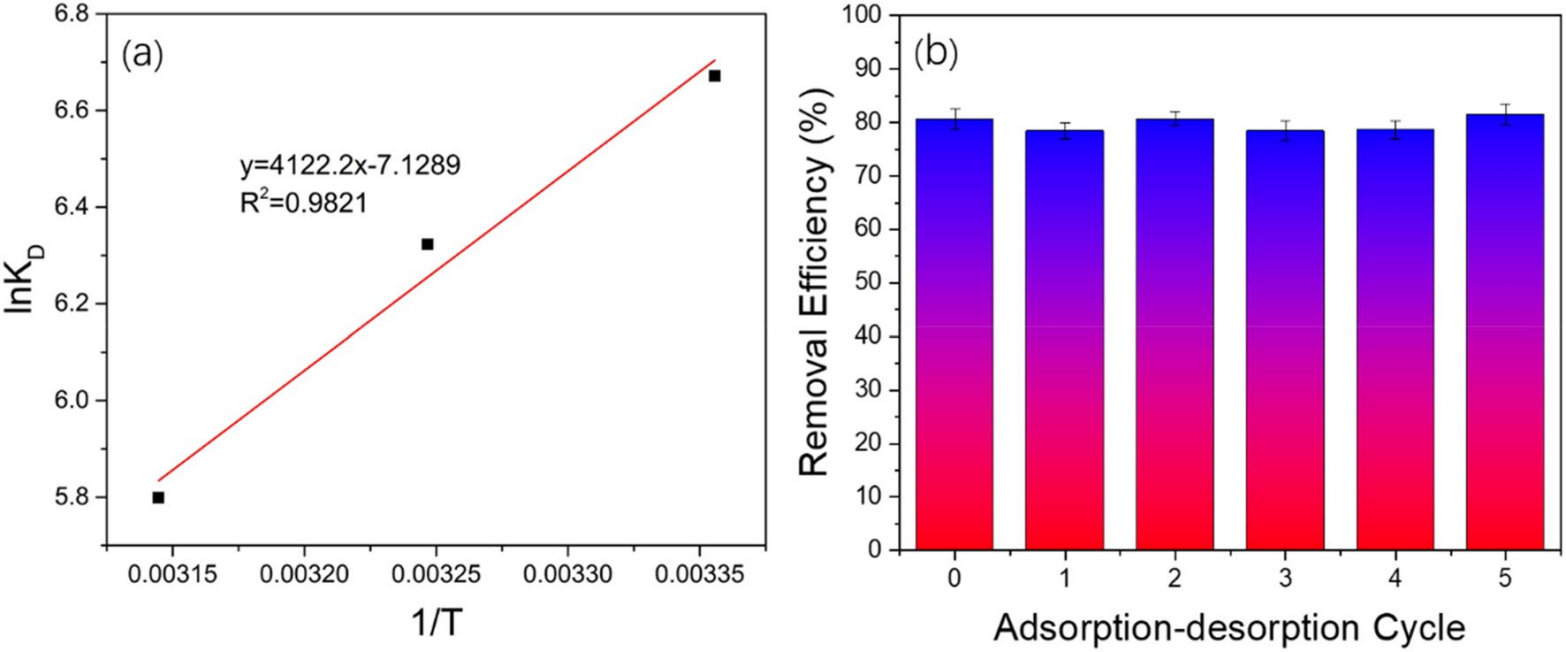
(**a**) Linear fitting for the thermodynamic parameters for the Sb(III) sorption onto GAD. (**b**) Adsorption–desorption cycle of GAD.

The value for K_D_ was calculated using Formula (12) based on the date of the experiment for the removal of Sb(III) by the GAD hydrogel beads. Then graphs of lnK_D_, vs, 1/T, ΔH, and ΔS were calculated using Formula (11) and ΔG with Formula (10) and plotted in Fig. 7a.

As shown in Table 5, the enthalpy change ΔH of the adsorption reaction is −34.27 kJ/mol and is less than 0, therefore, the reaction was an exothermic reaction and consistent with the conclusion above. The entropy ΔS is also less than 0, indicating this reaction decreased in the direction of chaos. The Gibbs free energy ΔG values of the different temperatures are − 16.61 kJ/mol, − 16.01 kJ/mol, and − 15.42 kJ/mol. The absolute value of ΔG decreased with the enhanced reaction temperature. These results indicate that this adsorption reaction was spontaneous. The adsorption decreases with increase in temperature and molecules adsorbed earlier on a surface tend to desorb from the surface at elevated temperatures. The data showed that the sorption process is spontaneous and exothermic in nature and that lower solution temperatures favors Sb(III) ion removal by the GAD. The decrease in adsorption with increasing temperature, suggest weak adsorption interaction between biomass surface and the Sb(III) ion, which supports physisorption while the high reaction temperature did not support adsorption.

**Table 5:** Thermodynamic parameters for the Sb(III) sorption onto the GAD hydrogel beads.

T(K)	ΔG (kJ/mol)	ΔH (kJ/mol)	ΔS (kJ/(mol K)
298	− 16.61		
308	− 16.01	− 34.27	− 0.0593
318	− 15.42		

### Regeneration experiment

The recyclability of the adsorbents is an important indicator for evaluating the economics of the adsorbents in practical applications. A reliably recyclable adsorbent effectively lowers the cost of the field application^57^. Adsorbents with a high adsorption capacity are an important potential source of heavy metals, and they are widely used in industry. An ideal adsorbent usually has a long useful life and high adsorption efficiency. Generally, HCl, NaOH, EDTA, and inorganic salts are used as desorption reagents in regeneration experiments^58^. A 1 mol/L CaCl_2_ solution was used in the adsorption–desorption experiment. As shown in Fig. 7b, the Sb(III) adsorption efficiency of GAD is 80% and after 5 times recycling through adsorption desorption, this excellent adsorption performance is maintained. Compared to the initial adsorption experiment, the adsorption efficiency does not change, indicating the GAD beads are good sorbents for this reaction.

### Column experiment

**Figure 8:**
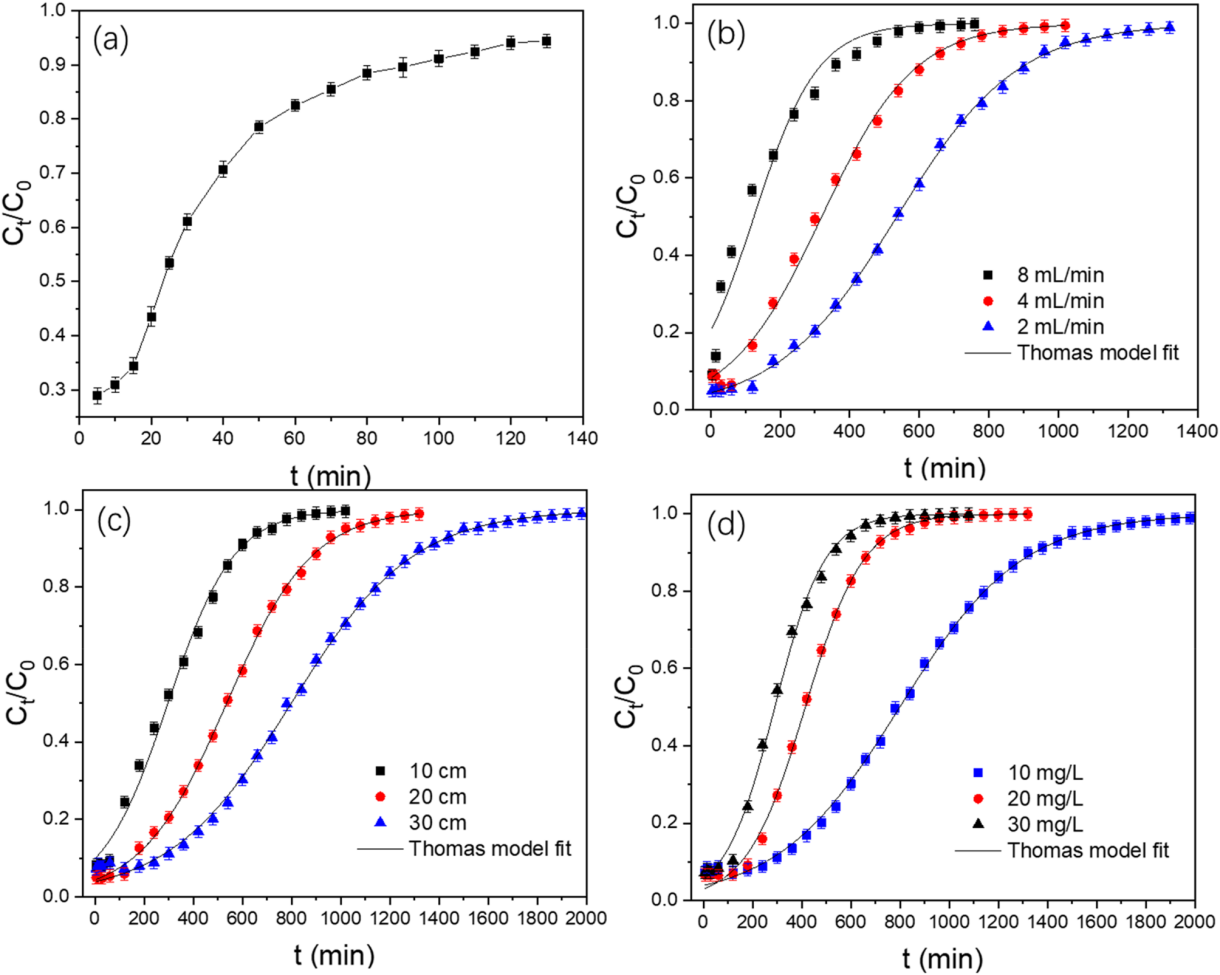
(**a**) Breakthrough curves for raw sand (blank control), bed depth, 20 cm; Sb(III), 10 mg/L, flow rate 8 mL/min. (**b**) Breakthrough curves for GAD with different hydraulic loading rates, 20 cm, 10 mg/L. (**c**) Breakthrough curves for GAD with different bed depths, 2 mL/min, 10 mg/L. (**d**) Breakthrough curves for GAD with different feed concentrations, 2 mL/min, 30 cm.

The different application scenarios to show the adsorption performance of the GAD beads by the column experiments can provide useful information for the design of a full-scale treatment. The breakthrough curves are graphed by plotting C_t_/C_0_ versus t (Fig. 8). C_t_ is Sb(III) concentration in the column outflow, and C_0_ is the Sb(III) concentration in the column inflow. The column adsorption breakthrough capacity was calculated using the methodology developed by Treybal^43^, which is expressed as

13 $$ {\text{breakthrough}}\;{\text{capacity}} = \frac{breakthrough\;time \times flow\;rate \times feed\;concentration}{{mass\;of\;the\;adsorbents\;in\;bed\left( g \right)}} $$

A 1 cm sand layer was placed in the top and bottom of the GAD filter to ensure the uniform flow of the column and ensure the contact between the GAD beads and antimony-containing effluents. A pure sand filter was used as the control blank in the column experiment to determine the role of GAD in adsorption. The leakage points and the breakthrough point occurred when C_t_/C_0_ was equal to 0.1 and 0.9, respectively. The following experiment parameters were used: the bed depth was 20 cm; the initial concentration of Sb(III) was 10 mg/L; the flow rate was 8 mL/min; the reaction temperature was 298 K; and the pH of the solution was 6. Figure 8a shows the sand filter has no obvious adsorption effect on Sb(III). The value for C_t_/C_0_ was only 50% after 20 min and less than 10% after 90 min. Only a small amount of sand was used in the experiment, so it did not affect the Sb(III) transport.

#### Effect of the flow rate

The effluent of the flow rate of the column is an important factor affecting the hydraulic retention time of the system. An excessively fast flow rate may cause the pollutants and the adsorbents to be in insufficient contact, resulting in an unsatisfactory removal efficiency. However, too slow of a flow rate may release pollutants into the solution, reducing the effectiveness and utilization of the adsorption device. Therefore, the flow rate of the column is an important parameter for the design and application of the column system (Fig. 8b). In the different flow rates (8 mL/min, 4 mL/min, and 2 mL/min), the time needed for the column to reach the breakthrough point increases with the increasing flow rate. When the flow rate decreases from 8 to 2 mL/min, the time needed for the column to arrive at the breakthrough point increases from 5 to 150 min, and the time needed for the column to reach the breakthrough point increases from 360 to 960 min. When the flow rate increases, the breakthrough curve gradually steepens. The contact time between the Sb(III) ions and the GAD correspondingly is reduced due to the decrease of the hydraulic retention time, and the dynamic removal efficiency is lowered.

#### Effect of the bed depth

Different bed depths affect the contact time of the contaminated solution in the system. When the adsorption reaction is not strong, increasing the contact time helps to improve the overall removal efficiency of Sb(III) from the solution. An increase in bed depth also leads to an increase in the mass of the adsorbent in the apparatus, providing a relative increase in the dose in this reaction. Figure 8c shows that the time needed for the column to arrive at the leakage point and breakthrough point increases with the height of the bed depth. When the bed depth increases from 10 to 30 cm, the time needed for the column to reach the leakage point increases from 60 to 300 min, and the time needed for the column to arrive at the breakthrough point increases from 600 to 1320 min. The increased depth quickly enhances the adsorption sites and hydraulic retention time. Both enhance the overall adsorption reaction and performance of the column and soften the breakthrough curve^71^. However, an excessively high bed depth leads to a higher local head loss and increases in the material cost and cost of the entire system. It is necessary to not only consider the purification efficiency of the wastewater, but also the running cost of the treatment method, so the bed depth is important.

#### Effect of the initial concentration

The initial concentration is also important for the optimal removal efficiency of the column system. To study the effect of the initial concentration in the column, experiment was carried out with initial concentrations of 10 mg/L, 20 mg/L, and 30 mg/L, respectively. The increase in the initial concentration of the pollutant in the column led to a higher adsorption efficiency in the device, which caused the limited adsorption sites in the adsorption column to be rapidly occupied by the pollutants in the high-concentration effluents, thereby affecting the column leakage and breakthrough^72^. Figure 8d shows that when the initial concentration increases, the utilization of the column decreases. At 10 mg/L, 20 mg/L, and 30 mg/L, the leakage time of the column is 300 min, 180 min, and 120 min, respectively, and the breakthrough time is 1320 min, 690 min, and 540 min, respectively. When the concentration of antimony-containing effluents increases, the concentration gradient between the solid and liquid in the system increases, so that the reaction proceeds in a direction favorable for adsorption. Therefore, the adsorption sites in the adsorbents are rapidly filled, causing the column to rapidly reach the leakage and breakthrough points.

#### Mathematic modeling

The Thomas model assumes a flat push flow during the column experiment, and it is a mathematical model widely used for column experiments^73^. Its expression is as follows:

14 $$ \ln \left(\frac{C0}{{Ct}} - 1\right) = \frac{kThq0m}{Q} - kThC0t $$

where k_Th_ is the Thomas model constant (mL/min mg); q_0_ is the amount of Sb(III) adsorbed per g of the adsorbent at equilibrium; C_0_ (mg/L) is the inlet Sb(III) concentration; C_t_ (mg/L) is the outlet concentration at time t; m (g) is the mass of the adsorbents; and Q (mL/min) is the flow rate. The plots of ln[C_0_/C_t_) − 1] versus time (t) result in a straight line, where the slope is k_Th_ and the intercept is q_0_ (figure not shown). The parameters of the Thomas model are shown in Table 6.

**Table 6:** Thomas model parameters at different conditions.

C_0_ (mg/L)	H (cm)	Q (mL/min)	k_Th_ (mL/(mg min)	q_0_ (mg/g)	R^2^
10	20	8	0.980	2.59	0.98
10	20	4	0.770	2.97	0.99
10	20	2	0.580	2.39	0.99
10	10	2	0.780	2.74	0.99
10	20	2	0.580	2.39	0.99
10	30	2	0.385	2.31	0.99
10	30	2	0.385	2.31	0.99
20	30	2	0.380	2.42	0.99
30	30	2	0.317	2.63	0.99

Figure 8 and Table 6 show that the Thomas model has a good fit with the data from the column experiment, and the correlation coefficients are above 0.98 under different conditions. The adsorption capacity (q_0_) of Sb(III) is in the range 2.31–2.97 mg/g, which is a little less than the maximum adsorption capacity in the batch experiment (q_max_ = 7.67 mg/g). The reason for this is that the contact time between Sb(III) and the adsorbent is limited and leads to the reaction occurring predominantly through a physical process. Chemical adsorption needs a longer contact time. Even so, the adsorption performance of GAD in the column is still better than that of other adsorbents, such as bentonite (0.56 mg/g)^14^, MWCNTs (0.33 mg/g)^15^, and iron oxide coated sand (2 mg/g)^65^. When the initial concentration increases, the Thomas model constant K_Th_ decreases, which is due to the increase in the concentration gradient between the solid and liquid, thereby increasing the driving force of the mass transfer and improving the chance of contacting Sb(III) with the adsorption sites in GAD. Hence, the adsorption efficiency is enhanced^74^. When the bed depth increases from 10 to 30 cm and the flow rate is reduced from 8 to 2 mL/min, K_Th_ is gradually reduced. The actual results of the removal efficiency of outlet and time needed for column leakage show the utilization of the column was enhanced. This result indicates that the increased bed depth, decreased flow rate, and increased initial concentration are conducive to the effective sorption of Sb(III) ions on GAD.

#### The distribution of Sb(III) at the leakage point

**Figure 9:**
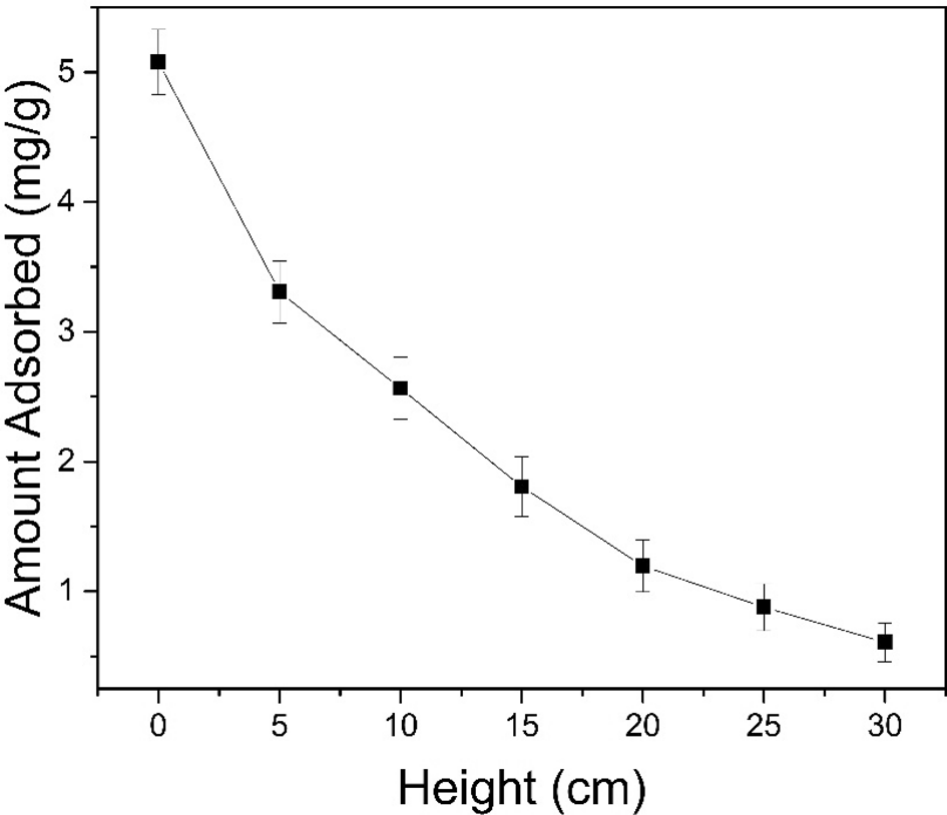
The distribution of the Sb(III) sorption GAD at different heights (from the column base) at the point of column leakage.

To determine the Sb(III) distribution at different heights in the column at the leakage point, we used the upward flow of the water intake from the bottom to the top. The experiment parameters were as follows: the bed depth was 30 cm, the flow rate was 2 mL/min, the initial concentration of Sb(III) was 10 mg/L, the reaction temperature was 298 K, and the pH was 6. The solution in the column was drained and the flow stopped when C_t_/C_0_ was 0.1. The samples of GAD (about 2 g of dry weight) were taken at the bed depths of 30 cm, 25 cm, 20 cm, 15 cm, 10 cm, 5 cm, and 0 cm. Then, the samples were immersed in 1:1 HCl for 24 h. This antimony-containing solution was monitored by flame atomic absorption spectroscopy after being immersed in hydrochloric acid and filtered with a 0.45-μm membrane. Figure 9 shows that the distribution of Sb(III) at different heights is not uniform, and the Sb(III) content decreases with the increase in height. The Sb(III) content is only 0.61 mg/g at a height of 30 cm, and 5.10 mg/g at a height of 0 cm, slightly less than the theoretical maximum adsorption capacity.

## Conclusions

The GAD bead, which had the benefits of the large specific surface of graphene and the low cost and reasonable biosecurity of sodium alginate, was synthesized. This novel material has been enhanced through the features of its two main constituents, which include the adsorption stability, acid and alkali resistance, thermal stability, and solid–liquid separation. The adsorption characteristics of GAD were further investigated by a range of experiment conditions, adsorption kinetics and isotherms, and thermodynamic parameters. The removal efficiency of GAD-adsorbed Sb(III) ions in solution was about 80% at pH 3–9, and the removal efficiency was only affected by a high concentration of NaCl that competed with Sb(III) for the adsorption sites on the surface of GAD. This adsorption fit well with the pseudo-second-order kinetic model, the Langmuir isotherm, and the Freundlich isotherm, which all describe the mechanism of the Sb(III) sorption to GAD. We speculated that the adsorption mainly consists of a physical process. The maximum adsorption capacity was 7.67 mg/g at 298 K, higher than many other common adsorbents. The thermodynamic parameters indicate that this adsorption reaction was exothermic and performed poorly at high temperatures. GAD was robust and recyclable up to five times, and the removal efficiency of Sb(III) was maintained at around 80%. The GAD beads behaved well during adsorption in the column experiments, and the Thomas model produced a strong fit to the experimental data under different conditions. GAD is a promising material for water treatment. It can be used for Sb contamination, but has the potential for broader applications.
